# Treatment of Capitellum and Trochlea Fractures Using Headless Compression Screws and a Combination of Dorsolateral Locking Plates

**DOI:** 10.7759/cureus.13740

**Published:** 2021-03-06

**Authors:** Shiro Yoshida, Kensuke Sakai, Kenjiro Nakama, Mitsuhiro Matsuura, Shingo Okazaki, Kotaro Jimbo, Masahiro Shirahama, Naoto Shiba

**Affiliations:** 1 Department of Orthopedic Surgery, Kurume University School of Medicine, Kurume, JPN; 2 Department of Orthopedic Surgery, St. Mary's Hospital, Kurume, JPN

**Keywords:** capitellum and trochlea fracture, coronal shear fracture, distal humerus, posterior comminution, lateral epicondyle fragment, dubberley

## Abstract

Introduction

This study aimed to evaluate the clinical outcomes of 16 patients with capitellum and trochlea fractures that were treated using isolated headless compression screws or a combination of dorsolateral locking plates and anterior-to-posterior screws. We also investigated the presence of lateral epicondyle fragments because this fragment is especially important when making decisions regarding the surgical approach and implants.

Materials and methods

We conducted a retrospective analysis of 16 patients with capitellum and trochlea fractures. Clinical, radiographic (based on CT scans), and elbow-specific outcomes, including the Mayo Elbow Performance Index (MEPI), were evaluated at a mean of 23.5 months postoperatively.

Results

The average MEPI scores in patients with Dubberley type A (non-posterior comminution) and type B (posterior comminution) fractures were 88 and 78, respectively (p=0.08). Headless compression screws were used in 10 cases of type A fracture and one case of type B fracture. A combination of dorsolateral locking plates and anterior-to-posterior screws was used in five cases of type B fracture. Hardware loosening was seen in one case of type B fracture with isolated screw fixation. The presence of a lateral epicondyle fragment was significantly associated with the type B group (6/6 patients; 100%). In contrast, patients in the type A group rarely had posterior comminution of the lateral epicondyle fragment (2/10 patients; 20%).

Conclusions

Capitellum and trochlea fractures with posterior comminution, which typically presented with lateral epicondylar fragments, were safely and effectively treated with a combination of dorsolateral locking plates and anterior-to-posterior screws through lateral approaches. Cases without posterior comminution were treated with headless compression screws with no complications. The Dubberley classification system provides helpful information to determine the fixation strategy.

## Introduction

Fractures of the humeral capitellum are relatively uncommon [1]. These fractures are caused by a direct force transmitted through the radial head. It provides a shearing and/or axial load to the capitellum and the trochlea [2]. McKee proposed a new type of fracture at the distal humerus, the “coronal shear fracture” [3]. It occurs with a fall from a height and results in the radial head impacting and shearing off the capitellum and the lateral ridge of the trochlea. Several studies of coronal shear fractures have received increasing recognition in the literature. Using headless compression screws is the gold standard treatment method and has shown mostly good-to-excellent results for all types of capitellum fractures [3-9]. Furthermore, capitellum fractures may be associated with lateral epicondyle fractures; in addition, more complex injuries with extension into the lateral trochlea, posterior comminution, or lateral condyle result in worse outcomes [7,10-12].

The purpose of this study was to report the clinical and radiographic outcomes of 16 patients with various coronal shear fractures of the capitellum treated with open reduction and internal fixation and to propose a treatment algorithm based on the fracture pattern with corresponding clinical outcomes. We also investigated the presence of lateral epicondyle fragments because this fragment is especially important when making decisions regarding the surgical approach and implants.

## Materials and methods

A total of 16 consecutive patients (3 men and 13 women; mean age: 49 years; age range: 11-78 years) who had sustained low-energy elbow injuries diagnosed as capitellum and trochlea fractures from the year 2001 to 2015 were included in the study. The most common mechanism of injury was a ground-level fall. All patients were followed for a minimum of 12 months (mean follow-up: 23.5 months). In all patients, a preliminary computed tomography (CT) scan with 3D reconstruction was taken of the fracture. All fractures were evaluated using the Dubberley subclassification: the fractures were classified as type A or B based on the presence of posterior comminution [11]. The mean time from injury to surgical treatment was 10 days (range: 5-19 days). The patients were subsequently followed clinically and radiographically, with subjective and objective outcome measures obtained. We performed anteroposterior and lateral elbow X-rays at one year post-operatively in all cases and evaluated capitellar atrophy. We defined capitellar atrophy as decreasing capitellum thickness compared to the contralateral side.

Elbow function was assessed using the Mayo Elbow Performance Index (MEPI) and the Patient-Rated Elbow Evaluation (PREE) [13]. This study was approved by our local Institutional Review Board was performed in accordance with the ethical standards as laid down in the 1964 Declaration of Helsinki and its later amendments.

**Figure 1:**
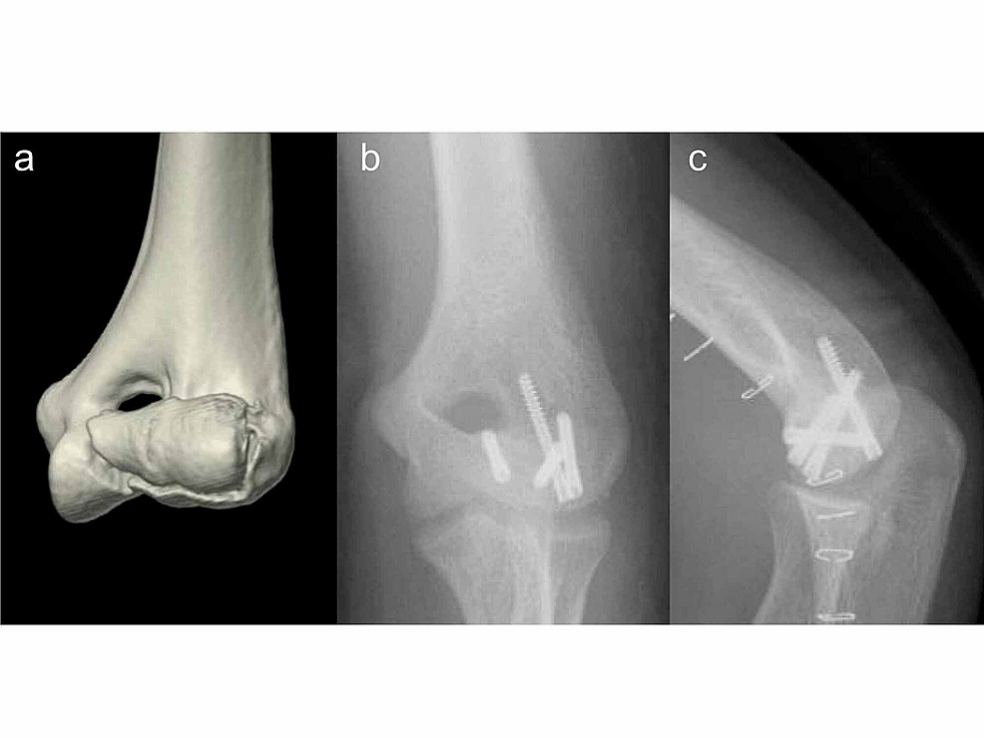
(a) Preoperative CT scan of a 15-year-old male patient with a Dubberley type 2A capitellum fracture. (b) Anteroposterior and (c) lateral X-ray views show the fragment fixation with headless bone screws.

**Figure 2:**
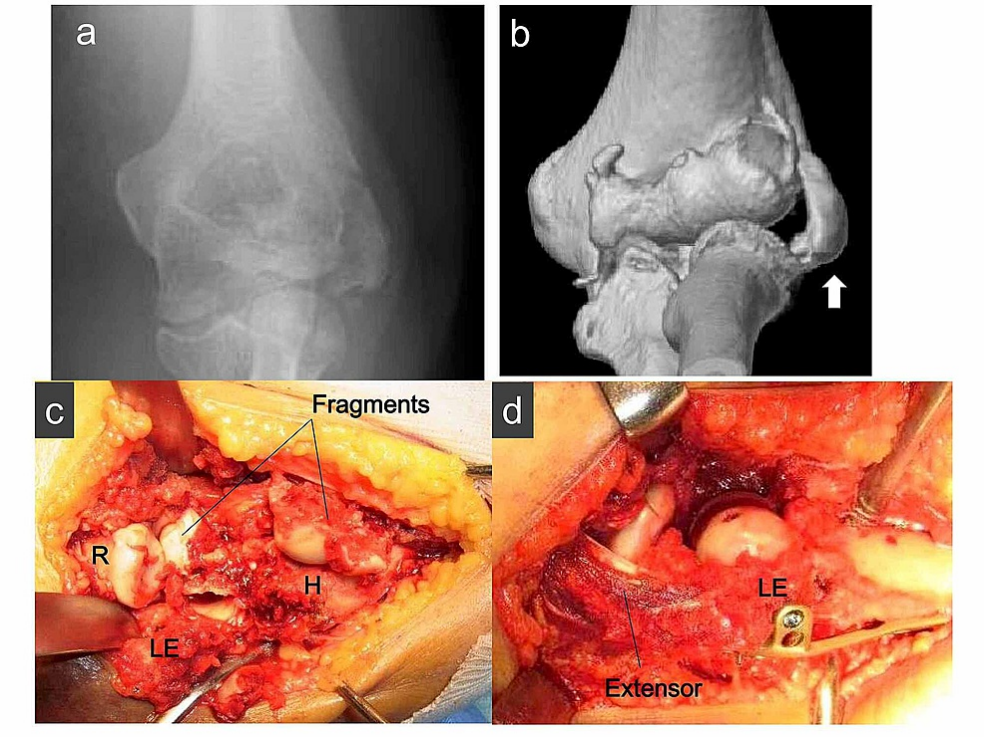
(a) Preoperative anteroposterior X-ray and (b) CT scan showing a lateral epicondyle fracture (white arrow) by Dubberley type 3B. (c) The lateral epicondyle fragment is retracted distally with the extensor origin. The elbow joint is exposed with the extensile Kocher approach. (d) Capitellum and trochlea fragments are fixed with anteroposterior headless screws after the lateral epicondyle fragment is fixed with a locking plate. R: radial head, H: distal humerus, LE: lateral epicondyle

**Figure 3:**
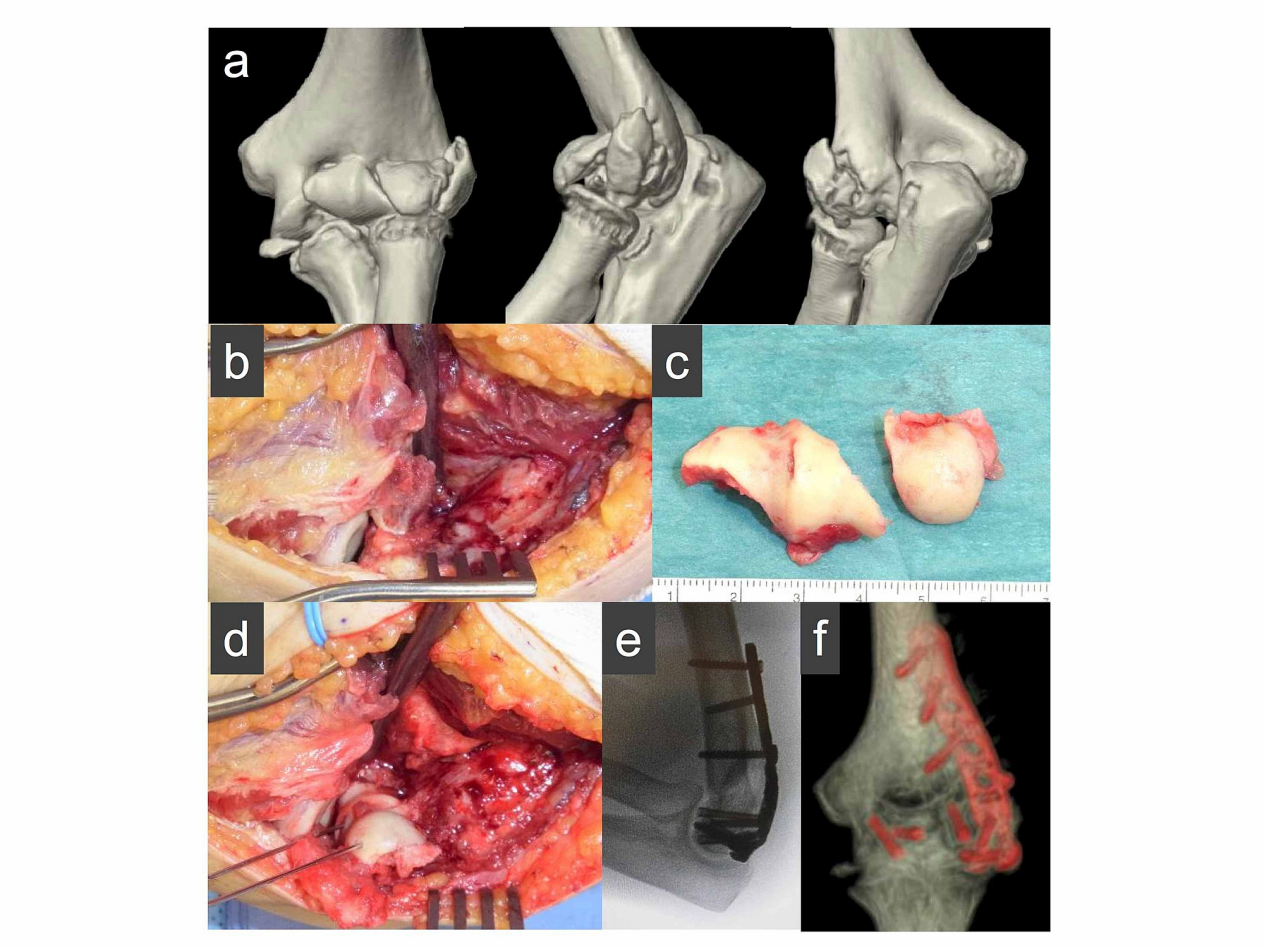
(a) Preoperative CT scan of a 72-year-old female patient with a Dubberley type 2B capitellum fracture with posterior comminution. An extensile Kocher approach was utilized, and the capitellum and trochlea fragments were removed (b, c) and re-affixed with provisional K-wires (d). (e) Postoperative X-ray shows adequate congruency of the radiocapitellar joint and (f) CT shows stable fixation with a posterior distal humerus locking plate.

In this study, 12 patients were treated with an extensile lateral approach (Kocher and Kaplan) and two patients were treated with an anterolateral approach when the middle of the posterior column was intact [14]. Two cases were indicated for a bilateral approach. In the first case, there was medial extension of the fracture; therefore, a medial approach was added in order to reduce the fracture accurately. In the other case, the capitellum was isolated medially to the elbow joint; therefore, an additional medial approach such as a flexor-pronator split was indicated, and the fragments were reduced correctly. All operations were performed with the patient in the supine position under general anesthesia. No olecranon osteotomy was performed. Headless compression screws were used in 10 cases of type A fracture and one case of type B fracture. Acutrak Mini headless compression screws (Acumed, Hillsboro, OR, USA) and small Herbert screws (Zimmer, Warsaw, IN, USA) were placed in an anterior-to-posterior direction and buried beneath the articular surface (Figure 1). In patients with lateral epicondylar fracture fragments, the lateral epicondylar fragment with the attached lateral collateral ligament (LCL) complex origin was reflected distally to enhance exposure (Figure 2). In posterior comminution cases, the lateral epicondylar fragment and capitellum fragment were provisionally reduced with a K-wire. One case was treated with headless compression screws and tension band wiring. In the other five cases, the locking plate was then placed on the lateral posterior aspect of the distal humerus to create a stable platform. After plating, the augmented anterior-to-posterior screws were placed and buried beneath the articular surface (Figure 3).

## Results

All fractures were united by the one-year follow-up appointment. The average MEPI score was 83.8 points (range: 60-100) with seven excellent, six good, and three fair ratings. The average total PREE score was 12.4 (range: 0-25) (p<0.04). Radiographically, there were five patients with a minimum of capitellum atrophy at their last follow-up appointment and one patient with osteoarthritis following hardware loosening. There were no instances of instability, non-union, or heterotopic ossification on the most recent radiographs. Statistically, the patients included significantly more women (13/16; 81%) than men (p<0.001). There were significant differences in MEPI scores between Dubberley type A and B. The average MEPI scores for type A and type B were 88 and 78, respectively (p=0.08). Posterior comminution was significantly associated with the final MEPI score. The incidence of lateral epicondyle fragments was significantly associated with the type B group (6/6 patients; 100%). On the other hand, patients in the type A group rarely had posterior comminution of the lateral epicondyle fragment (2/10 patients; 20%). One patient in this study had hardware loosening. This type B patient was treated with screws and tension band wiring fixation. The screws were backed out at first noted one year after the operation. The patient had postoperative stiffness with a 65° flexion contracture at the final postoperative visit (60 months). However, she did not have any difficulty in performing her daily work and desired no surgical intervention (Table 1).

**Table 1:** Comparison of type A and B fracture subgroups PPRE, Patient-Rated Elbow Evaluation; MEPI, Mayo Elbow Performance Index

	Type A (n=10)	Type B (n=6)	p-Value
PREE	8.2	20.9	0.04
MEPI score (points)	88	78	0.08
MEPI rating			
Excellent	5	2	
Good	4	2	
Fair	1	2	
Percentage of the presence of lateral epicondyle fracture	20% (2 of 10)	100% (6 of 6)	0.007

## Discussion

In this study, we focused on posterior comminution and lateral epicondyle fragments. We identified significant differences in MEPI scores between Dubberley type A and B. Posterior comminution was significantly associated with the final MEPI score. Our results showed that the MEPI score was significantly associated with posterior comminution. The standard Kocher surgical approach for preserving the LCL complex was most frequently used in the treatment of type A cases. Conversely, more aggressive approaches such as the extensile Kocher or Kaplan were used in the treatment of the majority of type B cases. Our results were similar to those reported in other studies [7,11,12]. Type A patients are more likely to be treated with a single Kocher approach with headless screw fixation, and better outcomes with a low complication rate are expected. Marinelli et al. suggested that type B patients should be treated with an extensile lateral or olecranon osteotomy approach [12]. However, in our study, we treated capitellum and trochlea fractures without olecranon osteotomy because the extensile lateral approach with the optional medial approach provides sufficient exposure in order to reduce and fix these fragments. The medial approach can be added intraoperatively when extended trochlea fractures are identified. We considered that the medial approach is less invasive than olecranon osteotomy and better avoids olecranon non-union.

In terms of patient age, the type B comminuted fracture pattern typically occurred in elderly patients and was associated with decreased range of motion of the elbow. On the contrary, the type A group also included elderly patients, but 4 of the 10 patients were teenagers who had fewer contractures and better bone quality without comminution. Our study demonstrated that coronal shear fractures were predominant in women (13/16). Mighell et al. suggested that an increased carrying angle could result in a larger contact force being impacted to the capitellum and trochlea during a fall with the elbow extension [7]. A second explanation of this trend could be account for the increased valgus carrying angle at the elbow in women compared to men [15-18]. This force could cause a higher risk of posterior comminution [7].

In this series, the presence of a lateral epicondyle fragment was significantly associated with the presence of posterior comminution, as 6/6 (100%) patients in the lateral epicondyle fragment group had some evidence of posterior comminution in contrast to only two patients who had a lateral epicondyle fragment in the presence of non-comminution (2/10 or 20%; p<0.007). These findings suggested that lateral epicondylar fragments signify the existence of potential posterior comminution.

Based on our experience, we have devised a treatment algorithm for capitellum and trochlea fractures. Internal fixation of capitellum and trochlea fractures requires perfect anatomical reduction and compression at the fracture site. The Kocher approach was chosen for isolated capitellum fractures. We chose to use anterior-to-posterior headless screws for Dubberley type A fractures. For fractures involving the capitellum and trochlea, the extensile lateral approach was useful, as it allowed wide exposure of the anterior elbow joint. On the contrary, for Dubberley type B fractures, one hardware loosening case demonstrated that dislocated headless screws were not able to fix the capitellum fragments, and we elected to use anterior-to-posterior screws in combination with posterior locking plates for the rest of the type B cases. This may provide more rigid fixation of the posterior column than isolated plate or screw fixation [19].

There were several limitations and weaknesses of our study. This was a retrospective study with a small sample size and a wide range of ages. However, these study characteristics reflect the rare nature of these injuries.

## Conclusions

In conclusion, we demonstrated that treatment strategy based on fracture patterns affects the outcomes in capitellum and trochlea fractures. Capitellum and trochlea fractures with posterior comminution, which often presented with lateral epicondylar fragments, were safely and effectively treated with a combination of dorsal locking plates and anterior-to-posterior screws through lateral approaches. No posterior comminution cases were treated with headless compression screws without complications. The Dubberley subclassification system helps us to provide helpful information to determine the best fixation strategy.
